# A RANDOMIZED CONTROLLED TRIAL OF METFORMIN FOR FRAILTY PREVENTION: STUDY DESIGN AND BASELINE CHARACTERISTICS

**DOI:** 10.1093/geroni/igz038.2517

**Published:** 2019-11-08

**Authors:** Sara E Espinoza, Nicolas Musi, Chen-pin Wang, Joel Michalek

**Affiliations:** 1 Barshop Institute for Longevity and Aging Studies, University of Texas Health Science Center, San Antonio, Texas, United States; 2 University of Texas Health Science Center, Texas, United States; 3 University of Texas Health Science Center, San Antonio, Texas, United States

## Abstract

Inflammation and insulin resistance are major predictors of frailty. Here we describe the study design of an ongoing double-blind, randomized controlled trial of metformin for frailty prevention. Subjects are adults aged 65+ years with pre-diabetes assessed by 2-hour oral glucose tolerance test (OGTT). Those who are frail (Fried criteria) are excluded. Participants are randomized to metformin (maximum dose of 2,000 mg/day) vs. placebo and followed for 2 years. The primary outcome is frailty (category and score); secondary outcomes are physical performance and function (short physical performance battery, 6-minute walk, lower extremity strength), systemic and skeletal muscle tissue inflammation, muscle insulin signaling, insulin sensitivity (insulin clamp), glucose tolerance (OGTT), and body composition (dual-energy x-ray absorptiometry). Safety assessments occur every 3 months; frailty, systemic inflammation, and OGTT are assessed at baseline and every 6 months, and insulin clamp with muscle biopsies are assessed at baseline and every 12 months. To date, 51 subjects have been randomized; 120 completers are planned. Mean age is 73.4 ±5.7 years, 43% are female, and 39% Hispanic. Mean BMI is 30.5 ±5.5 kg/m2, waist circumference is 105 ±13.1 cm, fasting glucose is 102.3 ±8.8 mg/dL, Hemoglobin A1c is 5.8 ±0.3, and glucose at 2 hours during OGTT is 168.5 ±20.4 mg/dL. Metformin is being examined in this study as a potential therapeutic agent to prevent frailty in older adults with pre-diabetes. Findings from this trial may have future implications for the screening and potential treatment of pre-diabetes in older patients with metformin for the prevention of frailty.

